# Gas Chromatography-Tandem Mass Spectrometry Method for the Selective Detection of Glycols and Glycerol in the Liquids and Aerosols of E-Cigarette, or Vaping, Products

**DOI:** 10.3389/fchem.2021.709495

**Published:** 2021-08-03

**Authors:** José J. Pérez, Clifford H. Watson, Benjamin C. Blount, Liza Valentín-Blasini

**Affiliations:** Tobacco Products Laboratory, Tobacco and Volatiles Branch, Division of Laboratory Sciences, National Center for Environmental Health, U.S. Centers for Disease Control and Prevention, Atlanta, GA, United States

**Keywords:** e-cigarettes, ethylene glycol, diethylene glycol, propylene glycol, glycerin, gas chromatography-tandem mass spectrometry

## Abstract

The long-term health effects of using e-cigarette, or vaping, products (EVPs; also known as e-cigarettes, electronic nicotine delivery systems, and vape pens) remain largely unknown. The inhalation of excipients, such as propylene glycol (PG) and glycerin (GLY), may have long-term health effects. In addition to the direct health effects of PG and GLY, glycerin-containing products can be contaminated with toxic ethylene glycol (EG) and diethylene glycol (DEG). To assess this issue, we developed a simple, versatile, high-throughput isotope dilution gas chromatography-tandem mass spectrometry method for quantifying these common excipients and contaminants. The method is applicable to both the liquid contents and machine-generated aerosols of EVPs. Our rigorous method validation demonstrates that the new method is specific, precise, accurate, and rugged/robust. The calibration range is linear from 0.1–7 mg for the excipients and 2.5–1,000 µg for the contaminants. These ranges encompass expected excipients levels in EVP e-liquids and their machine-generated aerosols and the relevant maximum residue safety limit of 1 mg/g, or 0.1% (w/w), for the contaminants. The calculated limits of detection for PG, GLY, EG, and DEG were determined as 0.0109 mg, 0.0132 mg, 0.250 µg, and 0.100 µg, respectively. The method was applied to the aerosol emissions analysis of 141 EVPs associated with the 2019 lung injury outbreak, and found typical levels of PG (120.28–689.35 mg/g of aerosol) and GLY (116.83–845.96 mg/g of aerosol) in all nicotine-containing products; PG (81.58–491.92 mg/g of aerosol) and GLY (303.86–823.47 mg/g of aerosol) in 13% of cannabidiol (CBD) products; PG (74.02–220.18 mg/g of aerosol) and GLY (596.43–859.81 mg/g of aerosol) in products with neither nicotine nor CBD; and none detected in tetrahydrocannabinol (THC) products. No products contained glycol contaminants above the recommended maximum residue safety limit.

## Introduction

Since entering the United States marketplace in 2007, the product landscape and popularity of e-cigarette, or vaping, products (EVPs) has expanded considerably (Olfson et al., 2019). Having evolved from their original cigarette-like appearance, a multitude of EVPs that differ in design and function are sold commercially and have gained widespread market acceptance (Gentzke et al., 2019; Huang et al., 2019). Despite their differences in design, nearly all EVPs operate based on the same basic principle of generating an aerosol from a liquid mixture of dissolved flavors and active ingredients (e.g., nicotine, THC, CBD). This solution, often referred to as the e-liquid, is resistively heated, and rapidly condensed into an aerosol as the user inhales air (puffs) through the device. Many e-liquids consist of mixtures with varying concentrations of propylene glycol (PG) and glycerin (GLY; also known as glycerol) diluents. The PG/GLY mixture serves as the excipient for efficient aerosolization and transfer of the active ingredient and flavor constituents from the EVP liquid to the user *via* the inhaled aerosol. Both PG and GLY are substances considered “generally recognized as safe” for human oral consumption by the United States Food and Drug Administration (USFDA) (21 C.F.R. § 182, 2020; 21 C.F.R. § 184, 2020). Both are used in a wide variety of consumer products including foods, medicines, cosmetics, and many types of personal care products. Although PG and GLY exposure *via* oral and dermal routes appear to be innocuous, little is known of the long-term health consequences of inhaled PG and GLY from sources such as EVPs (Callahan-Lyon, 2014). Although the EVP aerosol contains fewer known carcinogens than tobacco smoke, more data is needed to characterize their long-term health effects (National Academies of Sciences et al., 2018; Gotts et al., 2019).

A potentially compounding health risk associated with PG- and GLY-containing e-liquids is the possible contamination of raw materials with the toxic glycols, ethylene glycol (EG) and diethylene glycol (DEG) (Molever, 2010; Famele et al., 2015; Kavvalakis et al., 2015; Varlet et al., 2015) — known nephrotoxins and hepatotoxins which cause acute renal failure. Historically, many poisonings have occurred because of DEG contamination of GLY-containing products (Schep et al., 2009). The first documented case (1937) resulted in the deaths of more than 100 Americans across 15 states and prompted the enactment of the United States Food, Drug, and Cosmetic Act (FD&C Act) in 1938. More recent poisonings from DEG-contaminated products prompted the USFDA and the United States Pharmacopeia (USP) to issue guidance to manufacturers by recommending defined screening methods to ensure EG and DEG concentrations do not exceed the specified maximum residue safety limit of 1 mg/g, or 0.1% (w/w), of either substance in PG/GLY-containing products (USFDA Center for Drug Evaluation and Research, 2007; USP/NF, 2009). However, prior to 2016, the FD&C Act, and the guidelines and limits recommended and set by the USFDA and the USP, did not give the USFDA regulatory authority over EVPs and their components (including e-liquids); exposing the market to a wide array of products, including counterfeit and potentially contaminated products (Gottlieb, 2019). For example, in 2013, the USFDA issued alerts concerning possible DEG-contaminated EVPs and e-liquids imported from China entering the United States market (Peace et al., 2016). The USFDA ultimately gained regulatory authority over EVPs and their components in 2016; enabling scrutiny like that of other tobacco products, as well as the guidance laid out by the USFDA/USP for contaminants screening.

Despite the USFDA’s regulatory authority, no guarantees fully prevent the intentional or unintentional adulteration of do-it-yourself (DIY) (Cox et al., 2019), at-home e-liquid recipes prepared by persons attempting to make their own e-liquids, and/or the illegal introduction of counterfeit products. It is, therefore, important to remain vigilant in monitoring for these excipients and contaminants using accurate and reliable methodology that are fit for purpose. The USFDA/USP guidance describes a gas chromatography-flame ionization detector (GC-FID) method and offers an alternative procedure using a thin-layer chromatography (TLC) method (Kenyon et al., 1998). Other methods employ the use of derivatization (Molever, 2010; Kavvalakis et al., 2015) and/or do not target all four analytes mentioned thus far (Holloway et al., 2010; Varlet et al., 2015). Other methods have been developed to rapidly pre-screen samples prior to more thorough quantitative analyses by the GC-FID method, requiring two analytical runs for a possibly contaminated sample (Self, 2013; Peace et al., 2016). We describe here the development, validation, and application of a new, simple, sensitive, and selective isotope dilution gas chromatography-tandem mass spectrometry (ID-GC-MS/MS) method for the simultaneous quantitation and characterization of PG, GLY, EG, and DEG in the e-liquids and machine-generated aerosol emissions of EVP devices. This method was applied to the aerosol emissions analysis of EVPs associated with the 2019 outbreak of e-cigarette, or vaping, product use-associated lung injury (EVALI) (Blount et al., 2019; Centers for Disease Control and Prevention Office on Smoking and Health, 2019a; Blount et al., 2020; U.S. Food and Drug Administration, 2020).

## Materials and Methods

### Chemicals and Materials

Propylene glycol (PG; CAS# 57-55-6; ≥99.5%; meets USP testing specifications), glycerin/glycerol (GLY; CAS# 56-81-5; ≥99.5%), ethylene glycol (EG; CAS# 107-21-1; ≥99.9%; analytical standard), diethylene glycol (DEG; CAS# 111-46-6; 99.8%; pharmaceutical secondary standard; certified reference material), and isotopically labeled propylene glycol-*d*_8_ (PG-*d*_8_; CAS# 80156-55-4; isotopic purity: 98 atom % D, 99% chemical purity), glycerin/glycerol-*d*_8_ (GLY-*d*_8_; CAS# 7325-17-9; isotopic purity: ≥98 atom % D, ≥98% chemical purity), and ethylene glycol-*d*_4_ (EG-*d*_4_; CAS# 107-21-1; isotopic purity: 98 atom % D, 99% chemical purity) were obtained from Sigma-Aldrich (St. Louis, MO, United States). Isotopically labeled diethylene glycol-*d*_8_ (DEG-*d*_8_; CAS# 102867-56-1) was obtained from Toronto Research Chemicals (North York, ON, Canada). A second set of alternate-source, unlabeled PG, GLY, DEG, and EG standards were purchased from the U.S. Pharmacopeia (Rockville, MD, United States).

Methanol (MeOH; CAS# 67-56-1; HPLC grade; ≥99.9%) was obtained from Thermo Fisher Scientific (Waltham, MA, United States). Research grade helium (He) and ultra-high purity grade nitrogen (N_2_) gases were obtained from Airgas, Inc. (Hapeville, GA, United States). Deionized water (dI-H_2_O) was generated in-house using an Aqua Solutions model RODI-C-11BL ultrapure water (18 MΩ) purifications system (Jasper, GA, United States).

**FIGURE 1:**
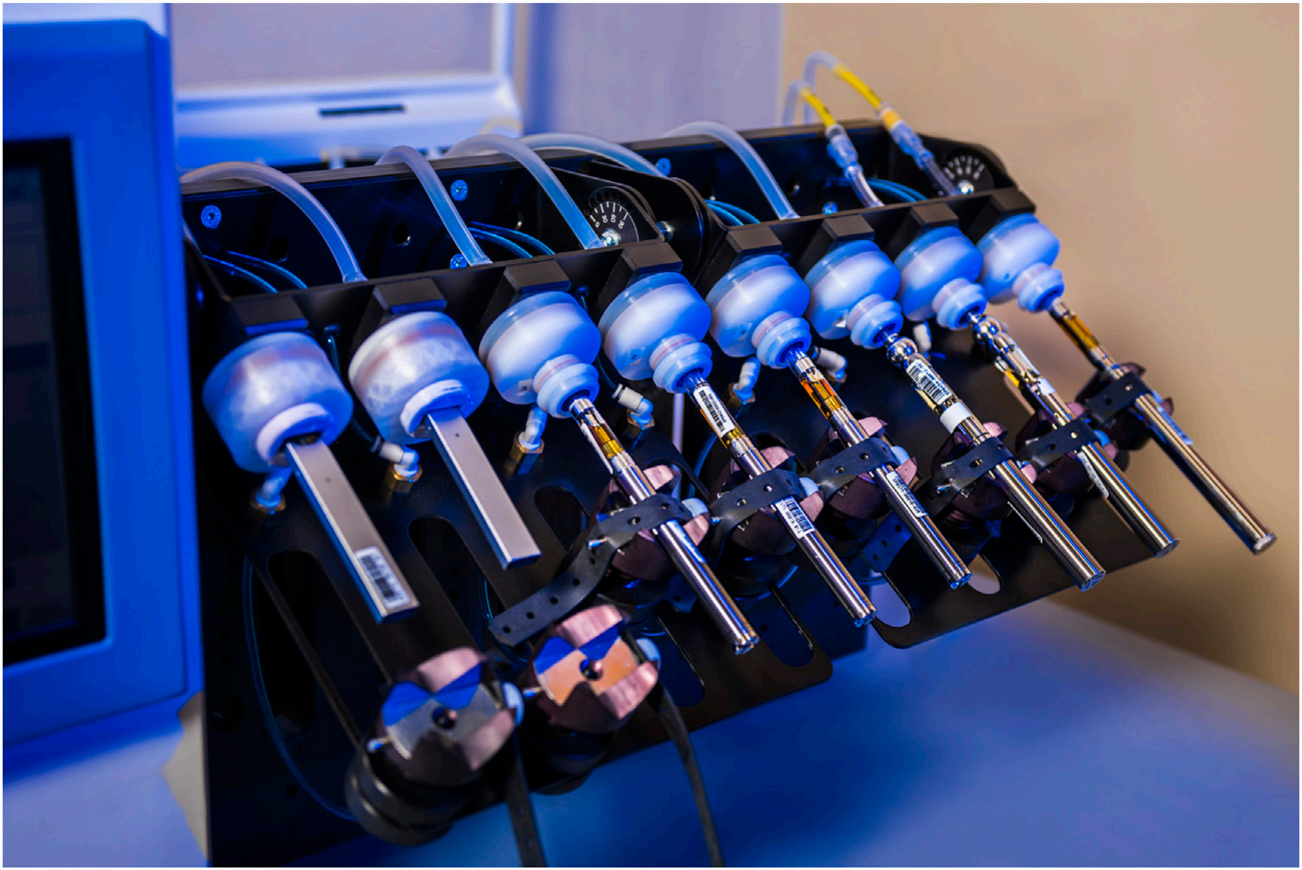
Experimental vaping setup of various EVPs analyzed. The two on the left demonstrate the custom-built holders and “lips” used for vaping products with mouthpieces of differing shape than those to the right which fit standard holders.

Cambridge filter holders used for collecting aerosol were purchased from Cerulean (Molins PLC, Milton Keynes, United Kingdom). Cambridge filter pads (CFPs; 44 mm) were purchased from Thermo Fisher Scientific (Waltham, MA, United States). Custom-made adapters (“lips”) used for vaping uniquely shaped device mouthpieces were fabricated in-house (see Figure 1).

### Standard and Quality Control Material Preparation

A Rainin AutoRep E Repeating Dispenser with corresponding syringe tips (Mettler-Toledo, Columbus, OH, United States) was used for positive displacement pipetting in the preparation of the following standard and quality control solutions. All solutions were stable and stored at room temperature.

#### Isotopically Labeled Internal Standards

Individual labeled internal standard (ISTD) stock solutions for each analyte were prepared in MeOH and combined to yield a single ISTD spiking solution with concentrations of 10 mg/ml (PG and GLY) and 500 µg/ml (EG and DEG). A 100 µl aliquot of this ISTD spiking solution was spiked into calibration standard solutions (described below) and all blanks, unknowns, and quality control (QC) samples.

#### Native Standards

Individual stock solutions for each unlabeled (native) analyte were prepared in MeOH and combined to prepare four stock solutions. Individual PG and GLY stock solutions were combined to prepare two stock solutions (A and B) with concentrations of 0.5 and 50 mg/ml, respectively. Individual EG and DEG stock solutions were combined to prepare two other stock solutions (C and D) with concentrations of 12.5 and 500 µg/ml, respectively. Stock solutions A–D were then used to prepare nine calibration standard solutions with concentration ranges of 0.01–7 mg/ml (PG and GLY) and 0.25–100 µg/ml (EG and DEG). Calibration standard solutions were all spiked with ISTD spiking solution upon preparation.

#### QC Materials

Additional mixtures were prepared to serve as matrix-based QC materials with low and high analyte levels spanning the calibration ranges of both analyte groups. The first QC pool mixture, QC_a_, was prepared by combining 32 g PG, 4 g GLY, 1 mg DEG, 1 mg EG, and 4 g dI-H_2_O to give 800 mg/g PG, 100 mg/g GLY, and 25 µg/g EG and DEG [density-adjusted PG, GLY, and dI-H_2_O composition corresponded to 80/10/10 PG/GLY/dI-H_2_O (v/v/v)]. A second QC pool, QC_b_, was prepared by combining 4 g PG, 32 g GLY, 240 mg DEG, 240 mg EG, and 4 g dI-H_2_O to give 100 mg/g PG, 800 mg/g GLY, and 6,000 µg/g EG and DEG [density-adjusted PG, GLY, and dI-H_2_O composition corresponded to 10/80/10 PG/GLY/dI-H_2_O (v/v/v)]. The PG and GLY levels between QC_a_ and QC_b_ pools were intended to account for possible variations of PG/GLY compositions in commercial products available on the market. The QC concentration levels were characterized to determine the mean concentrations and the 95th (1.96 σ) and 99th (2.96 σ) control limits by duplicate analysis of 20 samples of each QC level over at least 20 days. A 150 mg aliquot of each QC pool was extracted and analyzed concurrently with sample unknowns and the resulting QC data were compared to the established control limits to evaluate the validity of analyses using modified Westgard rules (Westgard et al., 1981; Caudill et al., 2008).

### Aerosols/Vaping

For aerosol analyses, samples were generated using a Cerulean CETI-8 e-cigarette vaping machine equipped with button activation switches (Cerulean, Richmond, VA) as shown in Figure 1. A soap bubble meter was used to calibrate and verify the vaping machine puff volume prior to use. Samples were vaped according to the standard conditions described in CORESTA Recommended Method No. 81 (CORESTA, 2015) (i.e., 55 ± 0.3 ml puff volume, 3 ± 0.1 s puff duration, 30 ± 0.5 s puff interval, with a square wave puff profile). The aerosol from 15 puffs (no clearing puffs) taken from vaped EVPs was collected on individual CFPs and gravimetrically determined (d = 0.00001 g) by mass difference of pre- and post-vaping CFPs for a given sample [i.e., trapped total particulate matter (TPM)]. The puff number could be varied, when necessary or appropriate, up to 50 puffs. Post-vaped CFPs were carefully removed from CFP holders and placed into 16 ml vials for extraction.

### EVP e-Liquids Sampling

EVP products vary significantly in their physical designs and, therefore, product-specific means were used to disassemble, when necessary, remove, and transfer approximately 100–150 mg liquid from a given product to a 16 ml vial for extraction. Sample masses of the liquids removed were recorded.

### Sample Preparation

Sample vials containing blanks, QCs, and post-vaped CFP and/or liquid unknowns were spiked with isotopically labeled ISTD spiking solution. Ten milliliters (10 ml) of MeOH was then added to each vial and all samples placed on an orbital shaker for 10 min at 160 rpm. An undiluted aliquot of extract was transferred to GC autosampler vials for EG and DEG analysis, whereas, for PG and GLY, a 10-fold dilution of sample extracts was done prior to analysis.

### Instrumental Analysis

Instrument parameters were optimized for chromatographic performance (i.e., injection, separation, peak shape, run time) and sensitive detection (i.e., collision energies, gain) of each analyte. An Agilent 7890B GC equipped with an Agilent GC Injector 80 autosampler and interfaced to an Agilent 7000C triple-quadrupole mass spectrometer (Agilent Technologies, Santa Clara, CA, United States) was used for GC-MS/MS analysis. Two separate injections were made for each sample: one for PG and GLY and one for EG and DEG analyses. A 1 µl aliquot of sample extract was injected onto a 15 m Agilent J&W DB-WAX capillary column with a 0.25 mm I.D. and 0.50-µm film thickness using a 400:1 (PG and GLY) and 10:1 (EG and DEG) split injection. Helium carrier gas was used at a constant flow of 1 ml/min. The injector and transfer line temperatures were set isothermally at 230 and 240°C, respectively. The initial column temperature, 100°C, was held for 1 min, increased to 180°C at 60°C/min, held for 0.5 min, and then increased to 240°C at 60°C/min and held for 3 min. The mass spectrometer was operated in positive electron ionization (+EI) mode and the resulting ions mass analyzed *via* selected-reaction monitoring (SRM). MS/MS parameters were as follows: electron energy −70 eV, source temperature 230°C, MS1 and MS2 quadrupole temperatures 150°C, electron multiplier voltage gain factor 10 (PG, DEG, EG) and 1 (GLY), mass resolution wide (MS1) and unit (MS2), collision cell quench gas (He) 2.25 ml/min, collision cell collision gas (N_2_) 1.5 ml/min.

Quantitation and confirmation ion transitions were monitored for each analyte and an isotopically labeled ISTD ion transition was monitored for the corresponding quantitation ion transition. The SRM transitions monitored, collision energies, dwell times, and transition type used for analysis are summarized in Table 1. Data acquisition and analysis were conducted using Agilent MassHunter Workstation software.

**TABLE 1:** SRM method specifications.

Analyte	Ion transitions	Collision energy (V)	Dwell time (msec)	Transition type
Propylene glycol (PG)	61.0→43.2	3	30	Quantitation
	45.1→43.2	15		Confirmation
	64.1→46.1	3		ISTD
Glycerol (GLY)	61.0→43.2	5	50	Quantitation
	61.0→61.0	1		Confirmation
	64.1→46.2	5		ISTD
Ethylene glycol (EG)	62.0→33.3	1	30	Quantitation
	62.0→31.3	5		Confirmation
	66.1→36.3	1		ISTD
Diethylene glycol (DEG)	76.1→45.2	8	30	Quantitation
	75.1→45.2	3		Confirmation
	82.1→49.1	8		ISTD

### Quantitation

Calibration curves were constructed from the linear regression of the calibration standards’ analyte-to-ISTD response ratios versus known standard concentrations, x, with 1/x weighting. The broad calibration concentration ranges used required weighting to improve the accuracy of the lower calibrators. For aerosol analysis, results (output in mg PG/GLY and µg EG/DEG) were normalized by TPM mass and/or puff count to determine analyte yields per gram of TPM (mg/g TPM for PG and GLY; µg/g TPM for EG and DEG) and/or per puff (mg/puff for PG and GLY; µg/puff for EG and DEG). For e-liquid analysis, results were normalized by e-liquid sample mass to determine analyte levels per gram of sample (mg/g for PG and GLY; µg/g for EG and DEG). A GLY-concentration-dependent correction factor for final calculation of EG measurements was also necessarily imposed (discussed below) according to the following equation:

$$EG_{\mathrm{corrected}}\mathrm{=E}G_{\mathrm{measured}}-\left(\mathrm{GL}Y_{\mathrm{measured}}\mathrm{\times 0}\mathrm{.0145}\right)$$ (1)

where:

• EG_corrected_ is the corrected amount of EG in µg
• EG_measured_ is the amount of EG in µg measured by the instrument
• GLY_measured_ is the amount of GLY in mg measured by the instrument
• 0.0145 is the average amount of EG in µg produced per mg of GLY within a given sample

### Method Validation

A full method validation was performed to confirm that the performance characteristics of the methods were accurate and fit-for-purpose. Figures of merit included analytical specificity, accuracy, dynamic range, linearity, limits of detection, matrix effects, precision, and ruggedness/robustness. A description of experiments and presentation of their results are described below in corresponding sections of the Results and Discussion section.

### Application: Products Associated With the 2019 EVALI Outbreak

The method was applied to measure glycols and glycerol in the aerosol emissions of 141 EVP products associated with the EVALI outbreak. These products were categorized as tetrahydrocannabinol (THC) containing products if THC >0.3% (w/w), nicotine containing products if nicotine >0.2% (w/w), and cannabidiol (CBD) products if CBD >1% (w/w) and THC <0.3%. A total of 194 samples was received; however, only 141 of these products were analyzed, as 35 products did not contain enough volume for testing and 18 products did not generate sufficient aerosol TPM deliveries (products that generated aerosols of less than 6.5 mg TPM per 15 puffs were considered inoperative and their data excluded). Samples were machine-vaped as described in *Aerosols/Vaping* and 15 puffs collected per product. All samples were handled following proper guidelines for the handling and analysis of potentially illicit drugs. Sample chain-of-custody was maintained and documented.

## Results and Discussion

### Figures of Merit

#### Analytical Specificity

**FIGURE 2:**
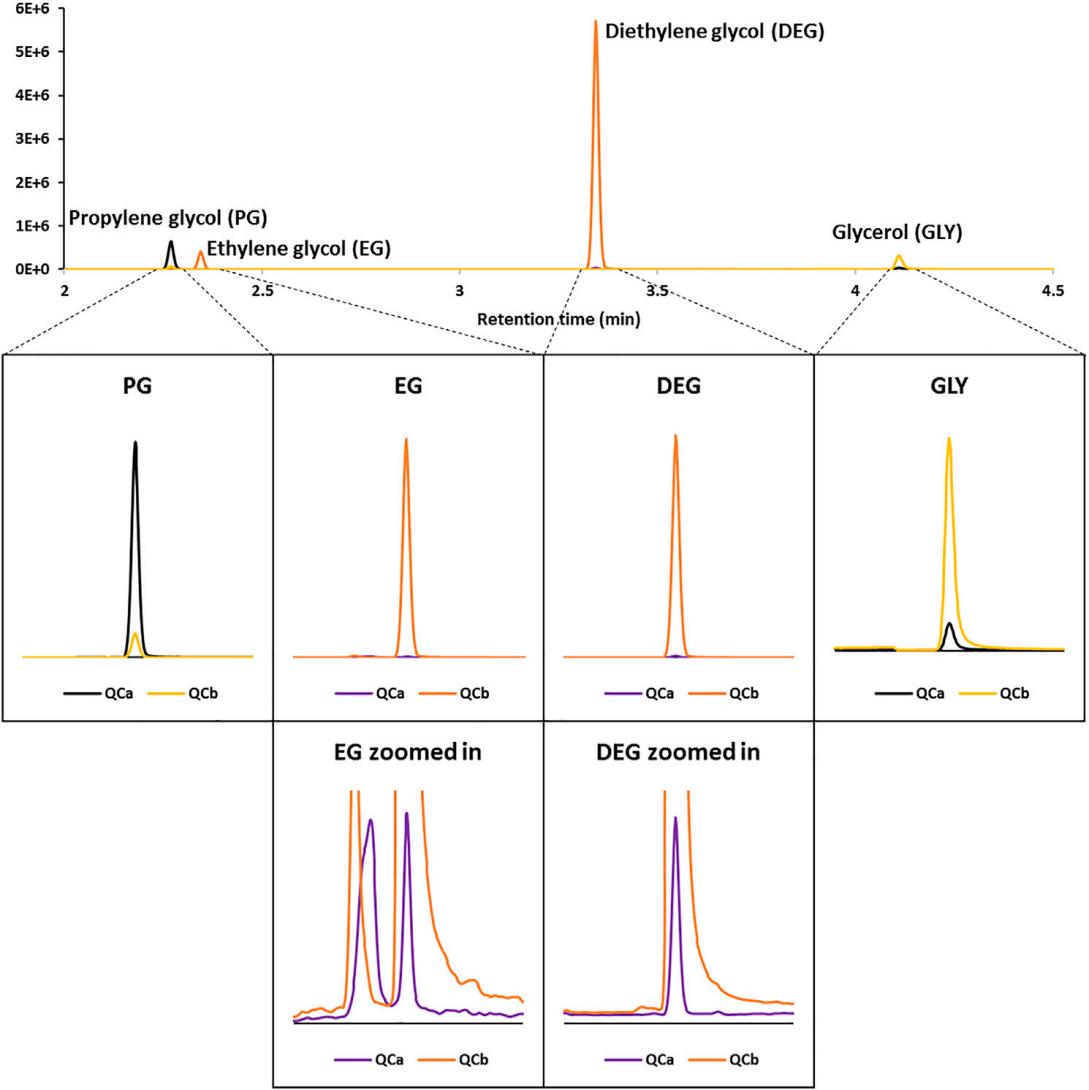
Overlaid selected-reaction monitoring (SRM) chromatograms of the two QC levels.

Specificity was demonstrated by baseline-resolved chromatograms and the absence of interfering matrix components in representative EVP samples. Figure 2 shows overlaid SRM chromatograms of the two QC levels. The use of isotopically labeled ISTDs also provided an additional level of retention time specificity. Despite their relatively simple structures and small masses, each analyte produced MS/MS spectra which allowed for the monitoring of distinct quantitation and confirmation ion transitions (Table 1). The only exception, GLY, had no additional product ion that could be used for confirmation. In this case, a pseudo-MS/MS ion transition was used by monitoring m/z 61 for both precursor and product ions with a low collision energy voltage. MS/MS response ratios between quantitation and confirmation ion transitions further increased method specificity.

#### Dynamic Range, Linearity, and Limits of Detection

LODs determined based on the method described by Taylor (1987) yielded calculated analyte LODs well below the intended purpose of the described method. Therefore, calibration (dynamic) ranges were selected such that the lowest reportable limit (lowest calibrator) was health relevant and application appropriate. The calibration range chosen for PG and GLY (0.1–70 mg) encompassed a full range of e-liquid compositions that may be possible in EVPs. The calibration range for EG and DEG (2.5–1,000 µg) was primarily chosen to encompass the maximum residue limits set by the USFDA (1 mg/g or 0.1%) and US Pharmacopeia Convention (620 µg/g or 0.062%). The concentration range implemented also encompassed potential EG and DEG levels below and above that of the specified limits for additional screening capabilities. Calibration curve linearity was confirmed by residuals analysis of the linear regression of seven separately prepared calibration curves with a coefficient of determination (R^2^) >0.98. Individual calibration curves yielded R^2^ > 0.99. A summary of method dynamic ranges, linearity, and calculated limits of detection can be found in Supplementary Table S1 of the Supplementary Material.

#### Accuracy and Matrix Effects

An accuracy study was conducted to evaluate potential concentration or matrix-based effects (i.e., PG/GLY content, samples with and without CFPs, and sample size) to ensure compatibility with: 1) the analysis of the diverse and dynamic market of EVPs; 2) both e-liquid (no CFP) and aerosol (with CFP) samples; and 3) sample sizes of varying degrees stemming from variable aerosol deliveries between products, product types, and/or machine vaping regimes.

Solutions with known concentrations of varying PG/GLY composition [100% PG, 75/25 PG/GLY, 50/50 PG/GLY, 25/75 PG/GLY, and 90/10 GLY/H_2_O (v/v)], each spiked with low, mid, and high concentrations of EG and DEG, were used as “matrix-matched” samples to assess accuracy. Accuracy results were acceptable at all concentrations and variations tested. All results were within 15% of their respective known concentrations, with most being within 5%. The accurate quantitation of matrix-based samples using a solvent-based calibration curve also indicated the absence of any matrix effects that could negatively affect measurements. These results show the applicability of the described method for accurate measurements in EVP e-liquid and aerosol samples of varying sample makeup. This recovery-based accuracy approach was necessary as no certified reference materials were available. A detailed summary of results can be found in Supplementary Table S2 (excipients) and Supplementary Table S3 (contaminants) of the Supplementary Material.

#### Precision

Method precision—evaluated as repeatability and intermediate precision—was assessed from the duplicate analysis of 20 samples of the QC materials (100 and 800 mg/g for PG and GLY; 25 and 6,000 µg/g for EG and DEG) over at least 20 different days (Supplementary Table S1). Repeatability was calculated as within-run variation of duplicates, while intermediate precision was calculated as the among-run, or total, variation. PG and GLY repeatability and intermediate precision ranged between 0.41 and 1.39% relative standard deviations (%RSDs) and 5.34–6.55% RSD, respectively. For EG and DEG, repeatability and intermediate precision ranged between 0.62 and 7.28% RSD and 5.33–13.3% RSD, respectively, with greater reproducibility at the higher concentration. Overall, the method precision was deemed acceptable with %RSDs <15%.

#### Ruggedness/Robustness Testing

Method ruggedness/robustness was tested by evaluating critical method parameters [i.e., matrix/excipients composition, CFP (aerosol) vs. no CFP (e-liquid), sample mass, extraction time, and extraction volume] that could potentially affect method performance and applicability. The PG-to-GLY composition ratio, the presence/absence of a CFP in sample, and the sample amount/mass were evaluated as part of the previously described accuracy experiments and showed no influence on the accuracy of results (Supplementary Tables S2, S3). Increasing extraction volumes and extraction times also showed no appreciable differences. For extraction volume, a ±20% change from the method-set parameter (10 ml MeOH) was tested and resulted in <3% difference in analytical results. Similarly, extraction time was also varied (15 and 30 min) from the method-set time of 10 min, and results showed <3% difference. Also tested was the vortexing (e-liquid) and repeat-inversion (10–15×) of samples (aerosol; CFPs fall apart if vortexed) rather than a defined extraction time at the defined 160 rpm. These results were also within 3% of results obtained under the prescribed method settings. These results indicate the efficient extraction/homogeneity of sample extracts prior to GC-MS/MS analysis.

#### Ethylene Glycol Correction Factor

**FIGURE 3:**
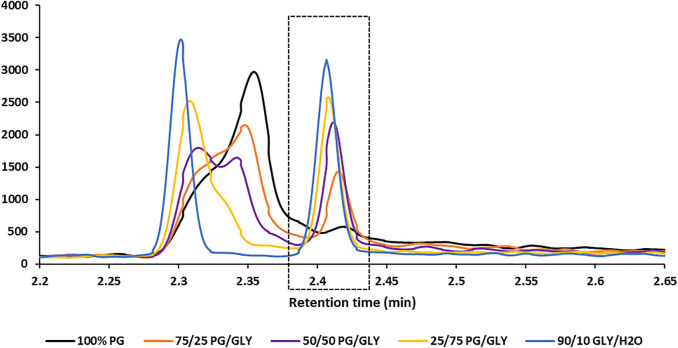
SRM chromatograms of the EG quantitation ion transition in five different blank matrix compositions illustrating the thermal degradation of GLY to produce EG (peak within dashed box). No EG is detected in a 100% PG matrix (no GLY; black trace); however, a corresponding increase in EG is observed with increasing GLY composition.

An EG moiety is found within the chemical structure of GLY. Because GLY is otherwise chemically and structurally stable (i.e., no decomposition or equilibrium), it is presumed that the thermal degradation of GLY within the heated GC injection port produced small, but detectable levels of EG; artificially elevating measured EG levels. Figure 3 illustrates the presence of an EG peak with the absence and increasing concentration of GLY from these mixtures. Measured EG levels from the analysis of blank PG/GLY mixtures [100% PG, 75/25 PG/GLY, 50/50 PG/GLY, 25/75 PG/GLY, and 90/10 GLY/H_2_O (v/v)] was used to determine the GLY-generated concentration of EG per milligrams of GLY (µg EG/mg GLY). A corresponding increased production and detection of EG was observed with increasing GLY concentration. A 0.0145 ± 0.0012 (SD) µg EG/mg GLY correction factor was determined and used for final calculation of measured EG measurements.

### Application: 2019 U.S. EVALI Outbreak

The method performed well in both hydrophilic (PG/GLY-based) and hydrophobic (oil-based medium chain triglycerides, vitamin E acetate, etc.) e-liquids obtained for the 2019 United States EVALI response, demonstrating its versatility. PG and GLY were detected at typical levels in nearly 43% (60 of 141) of the EVP aerosol samples analyzed, with no single product found to have had only PG or GLY alone. Table 2 summarizes measured concentration ranges (mg/g of aerosol) for these excipients in nicotine products (39 of the 60), CBD products (2 of the 60), and products with neither nicotine nor CBD (19 of the 60). PG (120.28–689.35 mg/g) and GLY (116.83–823.47 mg/g) were measured in all nicotine products. Although CBD products may be produced as either PG/GLY-based (hydrophilic) or oil-based (hydrophobic) liquids, only two CBD products were identified according to the criteria defined above (CBD >1%; THC <0.3%) and were both found to have PG (106.26 mg/g and 491.92 mg/g) and GLY (322.67 mg/g and 635.07 mg/g) as the excipients. Products void of either nicotine or CBD (i.e., no active ingredient) were also among the products analyzed with significant PG (74.02–443.72 mg/g) and GLY (491.33–859.81 mg/g) concentrations. Neither PG nor GLY were detected above LOD in any of the remaining 81 oil-based (hydrophobic), or THC-containing EVP samples [81 of 141 (57%)]. The absence of any detectable PG or GLY in the THC EVPs is consistent with the purportedly ubiquitous use of oil-based diluents [e.g., medium chain triglycerides (MCT) oil, coconut oil, vitamin E acetate, etc.]. Specifically, inhaled vitamin E acetate has been strongly linked with the EVALI outbreak (Blount et al., 2019; Centers for Disease Control and Prevention Office on Smoking and Health, 2019a; U.S. Food and Drug Administration, 2020) and shown to cause lung injury in mice (Bhat et al., 2020).

**TABLE 2:** Measured propylene glycol (PG) and glycerin (GLY) excipient concentration ranges by product type in EVALI-associated aerosol samples.

Product type	*n*	PG (mg/g)	GLY (mg/g)
Hydrophilic (PG/GLY)			
Nicotine	39	120.28–689.35	116.83–823.47
CBD	2	106.26–491.92	322.67–635.07
no Active ingredient	19	74.02–443.72	491.33–859.81
Hydrophobic (oil-based)^a^	81	<LOD	<LOD

a includes products containing THC and products with no active ingredient

For the glycol contaminants, no samples yielded results above the USFDA/USP specified relevant maximum residue safety limit of 1 mg/g (0.1% [w/w]). Trace signals of EG were detected in some samples (5%) but is most likely an analytical artifact of the previously discussed thermal degradation of GLY, as these samples contained GLY.

## Conclusion

The described dual-purpose ID-GC-MS/MS method provides accurate and precise quantitation of EVP excipients (PG and GLY) concentrations in e-liquids and their machine-generated aerosols, as well as screening and quantitation capabilities of the contaminants, EG and DEG, from a single sample. The method can be used to ensure EVPs containing PG/GLY mixtures comply with USFDA and USP standards. Application of the method toward an array of EVPs associated with the 2019 EVALI outbreak showed that the method is fit for its intended purpose and demonstrated its versatility by extended applicability to oil-based EVPs.

## Data Availability

The raw data supporting the conclusion of this article will be made available by the authors, without undue reservation.
