# Supplementary material for: Gas Chromatography-Tandem Mass Spectrometry Method for the Selective Detection of Glycols and Glycerol in the Liquids and Aerosols of E-Cigarette, or Vaping, Products
Source: Front Chem. 2021 Aug 3;9:709495. doi: 10.3389/fchem.2021.709495 (PMC8369378; doi:10.3389/fchem.2021.709495)
Supplement: Supplementary file 1 [file DataSheet1.PDF]

Supplementary Material

Table S1. Figures of merit.

**EXCIPIENTS**							
Analyte	LOD (mg)	Dynamic Range (mg)	Linearity (R^2; n=7)	Precision (%; n=20)			
				Repeatability		Intermediate Precision	
				100 mg/g	800 mg/g	100 mg/g	800 mg/g
PG	0.0109	0.1 – 70	0.997	1.03	0.41	5.58	5.34
GLY	0.0132		0.997	1.39	1.09	5.68	6.55
**CONTAMINANTS**							
Analyte	LOD (µg)	Dynamic Range (µg)	Linearity (R^2; n=7)	Precision (%; n=20)			
				Repeatability		Intermediate Precision	
				25 µg/g	6000 µg/g	25 µg/g	6000 µg/g
EG	0.250	2.5 – 1000	0.999	7.28	0.62	12.6	5.33
DEG	0.100		0.995	6.91	0.68	13.3	6.37

Table S2. Excipients method accuracy (% error).

Excipients composition (v/v)	Sample mass (mg)	Propylene glycol (PG)			Glycerol (GLY)		
		Concentration (mg/g)	CFP	no CFP	Concentration (mg/g)	CFP	no CFP
100% PG	50	975	2.3%	-3.9%	0	0.0%	0.0%
	100		0.1%	-9.8%		0.0%	0.0%
	200		-0.2%	-		0.0%	-
	400		-5.1%	-		0.0%	-
75/25 PG/GLY	50	695	1.9%	-3.7%	280	1.6%	1.6%
	100		1.3%	-8.3%		0.8%	-0.7%
	200		-0.8%	-		-0.8%	-
	400		-5.0%	-		-2.2%	-
50/50 PG/GLY	50	440	2.3%	-0.3%	535	-0.3%	-1.2%
	100		0.9%	-4.0%		-1.1%	-4.0%
	200		1.0%	-		-0.4%	-
	400		-1.8%	-		-3.8%	-
25/75 PG/GLY	50	210	5.4%	2.3%	765	0.4%	-3.5%
	100		4.3%	0.5%		-0.4%	-7.4%
	200		4.1%	-		-1.3%	-
	400		2.2%	-		-2.6%	-
90/10 GLY/H_2O	50	0	0.0%	0.0%	895	-2.8%	-3.6%
	100		0.0%	0.0%		-2.6%	-8.3%
	200		0.0%	-		-4.3%	-
	400		0.0%	-		-6.5%	-

Table S3. Contaminants method accuracy (% error) in varying excipients compositions.

Excipients composition (v/v)	Sample mass (mg)	Ethylene glycol (EG)						Diethylene glycol (DEG)					
		75 µg/g		375 µg/g		1875 µg/g		75 µg/g		375 µg/g		1875 µg/g	
		CFP	no CFP	CFP	no CFP	CFP	no CFP	CFP	no CFP	CFP	no CFP	CFP	no CFP
100% PG	50	-8.6%	-8.4%	1.4%	-0.4%	0.7%	0.1%	-3.3%	1.4%	2.8%	3.7%	6.5%	8.3%
	100	-5.9%	-3.5%	-0.5%	0.5%	0.4%	0.8%	0.9%	0.4%	2.6%	3.3%	5.2%	6.5%
	200	-5.9%	-	0.0%	-	0.9%	-	0.3%	-	2.7%	-	5.0%	-
	400	-1.6%	-	1.8%	-	0.7%	-	1.7%	-	5.2%	-	6.2%	-
75/25 PG/GLY	50	1.4%	11.7%	4.9%	3.2%	0.1%	-0.2%	-8.3%	-4.5%	3.3%	3.2%	7.3%	7.7%
	100	-1.5%	6.4%	1.3%	0.9%	-0.4%	-0.4%	0.0%	-1.2%	6.3%	4.6%	8.0%	8.4%
	200	2.2%	-	-1.0%	-	-1.7%	-	3.4%	-	5.8%	-	6.5%	-
	400	2.6%	-	-0.8%	-	-2.1%	-	3.5%	-	7.0%	-	6.1%	-
50/50 PG/GLY	50	1.6%	6.8%	-0.2%	3.6%	0.4%	1.7%	-10.4%	1.1%	0.1%	5.2%	2.8%	8.8%
	100	3.6%	1.8%	1.0%	2.5%	0.1%	-2.7%	-2.7%	-0.4%	2.7%	6.5%	2.9%	5.7%
	200	-0.8%	-	-0.9%	-	-3.0%	-	-1.2%	-	3.6%	-	2.4%	-
	400	-0.5%	-	-1.4%	-	-4.8%	-	0.6%	-	3.7%	-	1.8%	-
25/75 PG/GLY	50	6.3%	12.5%	12.5%	9.4%	10.9%	6.6%	-5.0%	-0.7%	4.1%	5.4%	5.6%	4.1%
	100	3.8%	14.1%	7.2%	6.7%	3.1%	5.5%	-3.3%	0.1%	0.2%	2.9%	-1.7%	3.9%
	200	4.6%	-	2.9%	-	2.7%	-	-2.5%	-	-1.6%	-	-1.4%	-
	400	4.8%	-	3.3%	-	1.4%	-	-1.2%	-	-1.7%	-	-2.1%	-
90/10 GLY/H_2O	50	4.2%	-7.6%	0.2%	0.8%	4.4%	2.7%	-3.5%	1.1%	-3.2%	-0.1%	-0.9%	2.4%
	100	-2.5%	0.6%	0.2%	2.9%	4.1%	2.0%	-6.5%	-2.1%	-5.4%	0.6%	-2.5%	1.1%
	200	-0.1%	-	1.9%	-	1.3%	-	-6.5%	-	-3.7%	-	-4.3%	-
	400	-1.5%	-	0.6%	-	-0.3%	-	-5.5%	-	-3.2%	-	-4.2%	-
